# Association between the p.Thr1406Asn polymorphism of the carbamoyl-phosphate synthetase 1 gene and necrotizing enterocolitis: A prospective multicenter study

**DOI:** 10.1038/srep36999

**Published:** 2016-11-11

**Authors:** Rob M. Moonen, Giacomo Cavallaro, Maurice J. Huizing, Gema E. González-Luis, Fabio Mosca, Eduardo Villamor

**Affiliations:** 1 Department of Pediatrics, Zuyderland Medical Center Heerlen, 6130 MB, The Netherlands; 2 Department of Pediatrics, Maastricht University Medical Center (MUMC+), School for Oncology and Developmental Biology (GROW), Maastricht, 6202 AZ, The Netherlands; 3 Neonatal Intensive Care Unit, Department of Clinical Sciences and Community Health, Fondazione IRCCS Cà Granda Ospedale Maggiore Policlinico, Università degli Studi di Milano, Milan, 20122, Italy; 4 Department of Pediatrics, Hospital Universitario Materno-Infantil de Canarias, Las Palmas de Gran Canaria, 35016, Spain

## Abstract

The p.Thr1406Asn (rs1047891) polymorphism of the carbamoyl-phosphate synthetase 1 (*CPS1*) gene has been linked to functional consequences affecting the downstream availability of the nitric oxide precursor L-arginine. L-arginine concentrations are decreased in preterm infants with necrotizing enterocolitis (NEC). In this multicenter prospective study, we investigated the association of the p.Thr1406Asn polymorphism with NEC in 477 preterm infants (36 cases of NEC) from 4 European neonatal intensive care units (Maastricht, Las Palmas de Gran Canaria, Mantova, and Milan). Allele and genotype frequencies of the p.Thr1406Asn polymorphism did not significantly differ between the infants with and without NEC. In contrast, the minor A-allele was significantly less frequent in the group of 64 infants with the combined outcome NEC or death before 34 weeks of corrected gestational age than in the infants without the outcome (0.20 vs. 0.31, *P* = 0.03). In addition, a significant negative association of the A-allele with the combined outcome NEC or death was found using the dominant (adjusted odds ratio, aOR: 0.54, 95% CI 0.29–0.99) and the additive (aOR 0.58, 95% CI 0.36–0.93) genetic models. In conclusion, our study provides further evidence that a functional variant of the *CPS1* gene may contribute to NEC susceptibility.

Necrotizing enterocolitis (NEC) remains a significant cause of morbidity and mortality in neonatal intensive care units. Although several predisposing factors have been identified, the exact etiology of NEC is yet elusive. The combination of genetic predisposition, immaturity of gastrointestinal motility, digestive ability, intestinal barrier function, immune defense and microcirculatory regulation accompanied by a strong likelihood of abnormal microbial colonization in the intestine, leads to a confluence of predisposing factors^1^,^2^,^3^,^4^,^5^,^6^,^7^,^8^,^9^.

Nitric oxide (NO) has received increasing attention in the pathophysiology of NEC, as it participates in the regulation of intestinal blood flow and plays a key role in the maintenance of mucosal integrity, intestinal barrier function, and post-injury intestinal repair^10^,^11^,^12^. NO is generated by NO synthase (NOS) during the enzymatic conversion of L-arginine to L-citrulline. The NOS substrate L-arginine is an essential amino acid for young mammals^13^,^14^. Metabolic and molecular studies indicate that the underdevelopment of intestinal arginine synthesis may be primarily responsible for remarkably low plasma arginine concentrations in preterm neonates^14^,^15^. Several studies demonstrated that plasma arginine concentrations are even more decreased in preterm infants with NEC^14^,^16^,^17^,^18^,^19^. Moreover, data from two small randomized controlled studies^16^,^20^, pooled in a meta-analysis^21^, suggest that arginine supplementation reduces the incidence of NEC in preterm infants.

Arginine is a urea cycle intermediate. The first step in the urea cycle occurs inside the mitochondrion and is catalyzed by the enzyme, carbamoyl-phosphate synthetase I (CPS1, **EC 6.3**.**4.16**)^22^. CPS1 deficiency in humans is a rare autosomal recessive inborn error of the urea cycle leading to hyperammonemia. Deficiency can be primary, due to mutations in the *CPS1* gene (OMIM 608307, HGNC:2323) or secondary, due to the lack of the essential cofactor N-acetyl-L-glutamate^22^. In addition, a number of functional single nucleotide polymorphisms (SNPs) have been identified in the *CPS1* gene^22^,^23^,^24^. One of those SNPs (p.Thr1406Asn also published as T1405N; rs1047891 formerly designated as rs7422339) has been linked to functional consequences affecting the downstream availability of urea-cycle intermediates, including L-arginine^22^,^23^,^24^. The SNP p.Thr1406Asn is a C-to-A nucleotide transversion (c.4217 C > A) in exon 36, which results in the substitution of asparagine (Asn) for threonine (Thr) in the critical N-acetylglutamate-binding domain^22^,^25^. The C-encoded Thr form of the polymorphism is considered the evolutionarily conserved version and the less frequent, A-encoded, Asn variant appears to be a relatively new, gain-of-function mutation^15^,^25^. It has been suggested that the A-allele may confer an advantage in terms of NO production, especially under conditions of environmental stress^15^. Previous studies demonstrated the association of the *CPS1* p.Thr1406Asn genotype with clinical situations where endogenous NO production is critically important, such as persistent pulmonary hypertension of the newborn^15^, pulmonary hypertension following surgical repair of congenital heart defects^26^, and hepatic veno-occlusive disease after bone marrow transplantation^23^.

Several years ago, we reported in a retrospective series of 17 preterm infants with NEC and 34 controls that patients with NEC showed an underrepresentation of the A-encoded variant of the p.Thr1406Asn polymorphism of *CPS1* 27. Those results suggested that the A-allele conferred protection against NEC and warranted confirmation using a prospective design and larger sample size. Herein we report the results of such study, involving 477 preterm infants (36 cases of NEC) from four neonatal intensive care units located in three different European countries (Spain, Italy, and the Netherlands). Since death in the first weeks of life is a competing outcome for NEC, we also analyzed the association of the p.Thr1406Asn polymorphism with the combined outcome NEC or death before 34 weeks of corrected gestational age(GA).

## Methods

### Patients

The study was approved by the Institutional Review Boards (IRBs) of the Maastricht University Medical Center (the Netherlands, registration number MEC 04–140), Hospital Universitario Materno-Infantil de Canarias (Las Palmas de Gran Canaria, Spain), Carlo Poma Hospital (Mantova, Italy) and Ospedale Maggiore Policlinico (Milan, Italy). The study was conducted according to institutional and IRB guidelines and regulations and registered in ClinicalTrials.gov Protocol Registration System (NCT00554866, ID 07-2-018, November 6, 2007). The manuscript was drafted according to the STROBE statement (http://www.strobe-statement.org/). All infants with a GA ≤ 30 weeks and birth weight (BW) ≤ 1500 g born between 1^st^ July 2007 and 30^th^ June 2012 and admitted to the level III neonatal intensive care unit of the above mentioned centers (Carlo Poma Hospital: inclusion until 31^st^ December 2010; Ospedale Maggiore Policlinico: inclusion from 1^st^ March 2011) were eligible for participation in the study. Written informed consent from the parents was obtained. As reported in a previous study, buccal cell samples were obtained from 96 healthy term infants (25 in Maastricht, 31 in Las Palmas de Gran Canaria and 40 in Mantova)^28^.

### Definition of clinical characteristics and outcomes

Data on clinical characteristics and outcomes were obtained from the medical records. GA was determined by the last menstrual period and early ultrasounds (before 20 weeks of gestation). Small for GA was defined as BW for GA below the sex-specific 10^th^ percentile. Chorioamnionitis was defined as every clinical suspicion of infection of the chorion, amnion, amniotic fluid, placenta, or a combination as judged by the obstetrician. Prolonged rupture of membranes was defined as rupture of membranes >24 hours before delivery. Prenatal exposure to a single course of antenatal steroids was defined as two doses of betamethasone administered 24 h apart and exposure to a partial course of antenatal steroids was defined as administration of a single dose of betamethasone <24 h prior to delivery.

NEC was defined as Bell stage II disease or greater. At the conclusion of the study, all cases of NEC were reviewed in a blinded fashion by a panel of 4 investigators of the study. Cases of spontaneous intestinal perforation (i.e., without pathologic evidence of NEC) were excluded from the investigation. Since it was considered that some infants who died in the first weeks may have developed NEC if they had survived, we also analyzed the composite outcome of NEC or death before 34 weeks of corrected GA.

Respiratory distress syndrome (RDS) was defined as requirement for oxygen supplementation or respiratory support due to tachypnea, grunting, nasal flaring, retractions, or cyanosis. Bronchopulmonary dysplasia (BPD) was defined as a supplemental oxygen requirement at 36 weeks of corrected GA to maintain oxygen saturation >90% 29. Arterial hypotension was defined as the need for volume expansion or inotropic support. Patent ductus arteriosus (PDA) was defined as a requirement for indomethacin or ibuprofen and/or surgical ligation. A diagnosis of sepsis required signs of generalized infection, a positive blood culture, and antibiotic therapy. Intraventricular hemorrhage (IVH) was classified by using the 4-level grading system^30^. Grade < 2 IVHs were not included in the analysis. Retinopathy of prematurity (ROP) was defined as stage II or higher.

### Samples and genotyping

Buccal cell samples for DNA testing were obtained with a sterile OmniSwab (Whatman), and collected in Eppendorf sterile PCR tubes and stored at −80 °C until further analysis. The samples obtained in Spain and Italy were transported on dry ice to Maastricht where all the analyses were performed. DNA was extracted using standard methods and stored at −20 °C until genotyping. A 214-bp fragment encompassing the p.Thr1406Asn polymorphism in exon 36 of the *CPS1* gene was amplified using polymerase chain reaction (PCR). Primers used were (forward) GCM357 5′-TAAATGCAGCTGTTTGCCAC-3′ and (reverse) GCM358 5′-GACTTGCAATCAAGTAAGGTGAAA-3′. The PCR mix consisted of 1X GeneAmp PCR Buffer II (Perkin-Elmer, Branchburg, NJ), 0.2 mM deoxyribonucleoside triphosphate (Pharmacia Biotech, Bridgewater, NJ), 1.5 mM MgCl2 (Perkin-Elmer, Branchburg, NJ), 250 nM of both primers, and 0.025 U/μL of AmpliTaq Gold (Perkin-Elmer, Branchburg, NJ). Thermocycling conditions started with an initial denaturation of 10 min 95 °C, followed by 35 cycles of 95 °C (45 s), 55 °C (45 s), 72 °C (45 s), and ended with a final extension step of 10 min at 72 °C. The PCR product was purified and directly sequenced using the reverse primer.

### Statistical Analysis

Sample size was calculated based on data from our previous study^27^. Given an expected population incidence of NEC of 5%, expected frequencies for CC homozygosity of 0.7 (NEC group) and 0.4 (control group), alpha level = 0.05, and power of 0.8, 440 infants were needed to detect an odds ratio (OR) significantly different from 1.

Categorical variables were expressed as counts or percentages and compared using the chi-square test. Continuous variables were expressed as mean (SD) if they followed a normal distribution and compared using unpaired, two-sided *t*-test. If not normally distributed, continuous variables were expressed as median values (interquartile range, IQR; 25^th^–75^th^ percentile) and compared using the Mann-Whitney U test. The Kolmogorov-Smirnov test was used to test for normal distribution of continuous data.

Differences in allelic frequencies and genotype distributions between the investigated populations, as well as Hardy–Weinberg equilibrium (HWE) for genotype distribution were assessed using a chi-square test. The Hardy–Weinberg law states that q^2^ + 2pq + p^2^ = 1, where p and q are allele frequencies in a two-allele system. Logistic regression analysis was used to compute the ORs and their 95% confidence intervals (CI) for NEC and the combined outcome NEC or death before 34 weeks of corrected GA based on genotype after accounting for the covariates which were significantly different between the groups and are known risk factors for developing NEC. Different genetic models were used to analyze the effect of the risk allele, including the general allelic (multiplicative or codominant model), dominant, recessive and additive models. Assuming a genetic penetrance parameter γ (γ > 1), a multiplicative model indicates that the risk of disease is increased γ-fold with each additional copy of the risk allele; an additive model indicates that risk of disease is increased γ-fold for the genotype with one copy of the risk allele and 2γ-fold for the genotype with two copies of the risk allele; a common recessive model indicates that two copies of the risk allele are required for a γ-fold increase in disease risk, and a common dominant model indicates that either one or two copies of the risk allele are required for a γ-fold increase in disease risk^31^. The major allele was considered as a reference and the interactions were tested in the different models by multivariable logistic regression model. All the statistical analyses were performed using IBM SPSS Statistics for Windows, Version 22.0. (IBM Corporation, Armonk, NY, USA) and conducted at the P < 0.05 level of significance.

## Results

### Patient characteristics

**Figure 1:**
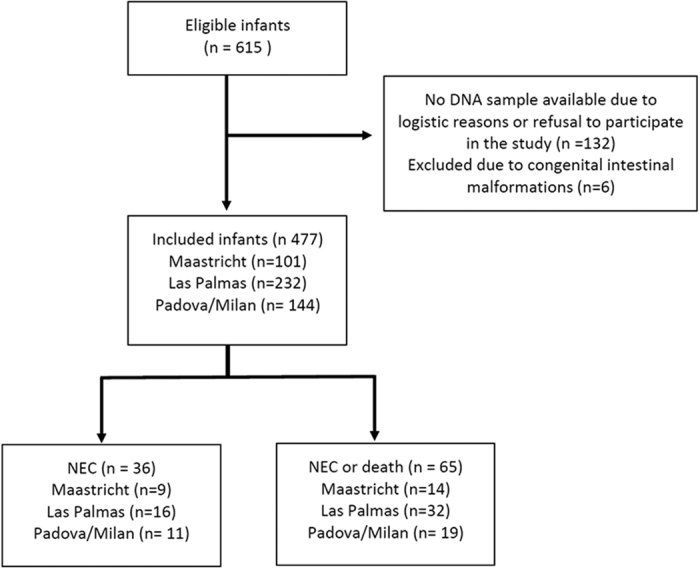
Flowchart of study participants.

From 615 eligible infants, 477 (36 with NEC Bell stage II or greater) were included in the study (Fig. 1). Stage II NEC was present in 23 infants and stage III in 13 infants. In 21 cases surgery was required. The median age at the onset of NEC was 20 days (range 4–87, IQR 12–31). Nine of the cases of NEC occurred in Maastricht (5 stage II NEC, 4 stage III NEC), 16 in Las Palmas (9 stage II NEC, 7 stage III NEC), and 11 in Italy (9 stage II NEC, 2 stage III NEC). Single intestinal perforation was present in 5 infants (1 in Maastricht, 4 in Italy). Demographic and clinical characteristics of the infants with and without NEC are shown and compared in Table 1. Mean GA, mean BW and median Apgar score at 1 min of infants with NEC were significantly lower than in infants without NEC. In addition, infants with NEC showed a higher incidence of vaginal delivery, mechanical ventilation, BPD, hypotension, IVH, PVL, PDA, ROP and mortality. We adjusted for GA, BW, Apgar score at 1 min, mechanical ventilation, hypotension, and PDA in the subsequent logistic regression analysis.

The demographic and clinical characteristics of the infants with and without the combined outcome NEC or death before 34 weeks of corrected GA are shown and compared in Table 2. Mean GA, mean BW and median Apgar score at 1 and 5 min of infants with NEC or death were significantly lower than in infants without the combined outcome. In addition, infants with the combined outcome NEC or death showed a higher incidence of vaginal delivery, mechanical ventilation, hypotension, IVH and PDA. We adjusted for GA, BW, Apgar score after at 1 and 5 min, mechanical ventilation, hypotension and PDA in the subsequent logistic regression analysis.

### Analysis of genotypes

Allele and genotype frequencies of the p.Thr1406Asn polymorphism in the total preterm population did not significantly differ from the allele and genotype frequencies observed in the population of 96 healthy term infants (*P* = 0.925, Table 3)^28^. The distribution of the genotypes p.Thr1406Asn did not fulfill Hardy-Weinberg criteria in the preterm population. The minor allele frequency (MAF) in the NEC group (0.208) was not significantly different from the MAF among the infants without NEC (0.303, *P* = 0.09; Table 4). The MAF in the NEC group with the combined outcome NEC or death before 34 weeks (0.200) was significantly different from the MAF among the infants without the outcome (0.311, *P* = 0.03; Table 5).

We further analyzed the effect of the CPS1 p.Thr1406Asn polymorphism on the occurrence of NEC and NEC or death under different genetic models. As shown in Table 4, logistic regression analysis could not detect any significant association between the p.Thr1406Asn polymorphism and NEC in any of the genetic models. In contrast, the dominant and the additive model showed a negative significant association of the A-allele of the CPS1 p.Thr1406Asn polymorphism with the combined outcome NEC or death before 34 weeks of corrected GA (Table 5). Finally, we analyzed the correlation of the *CPS1* p.Thr1406Asn polymorphism genotype with other neonatal outcomes. As shown in Table 6, we could not find any significant association.

## Discussion

This is one of the largest prospective studies investigating the association of a SNP with NEC. Our study could not detect a significant association between the p.Thr1406Asn genotype of the *CPS1* gene and the risk of developing NEC in very preterm infants (GA ≤ 30 weeks and BW ≤ 1500 g). However, when the combined outcome NEC or death before 34 weeks of corrected GA was analyzed, it was observed that the minor A-allele of the p.Thr1406Asn polymorphism was significantly less frequent in the group of infants who developed NEC or died than in those who survived without NEC. In addition, a significant negative association of the A-allele with the combined outcome NEC or death was found using the dominant and the additive genetic models.

In our previous retrospective case-control study, we examined the relationship between the *CPS1* p.Thr1406Asn polymorphism and the presence of NEC in preterm infants and we found that patients with NEC showed an overrepresentation of the C-encoded variant of the *CPS1* 27. Consequently, the A-encoded variant of the p.Thr1406Asn polymorphism was underrepresented in the infants with NEC. However, although the cases and the controls were well matched for GA and BW, when adjusted for these two known risk factors for NEC, the association between NEC and the p.Thr1406Asn polymorphism did not remain significant^27^. In the present prospective cohort study, we observed that the A-allele was less frequent in the infants with NEC in a proportion close to reach statistical significance (P = 0.09). Moreover, only one infant with NEC was homozygous for the A-allele. Nevertheless, none of the genetic models could demonstrate a statistically significant association between the p.Thr1406Asn genotype and NEC.

An important issue in designing studies involving high-risk patients is the selection of an appropriate primary outcome when death is a competing outcome^32^. In this situation, some patients will die before the outcome of interest can occur. For this reason, a composite outcome including death is often used when complications of prematurity such as BPD or NEC are studied^32^. Since the onset of ‘classical’ NEC takes place around the end of the third week of life^33^,^34^, we performed an additional analysis in which the infants with NEC were combined with the infants who died before 34 weeks of corrected GA. Interestingly, we observed that the minor A-allele was significantly less frequent among the infants with the combined outcome NEC or death. Moreover, the dominant and additive model demonstrated that the A variant of the p.Thr1406Asn polymorphism significantly decreased the risk of developing the combined outcome of NEC or death before 34 weeks of corrected GA. Thus, in other words, the minor A variant of the polymorphism might protect against this combined outcome.

In accordance with our results, it has been reported a protective role of the A-allele toward persistent pulmonary hypertension of the newborn^15^, pulmonary hypertension following surgical repair of congenital heart defects^26^, and hepatic veno-occlusive disease after bone marrow transplantation^23^. Moreover, the haplotype formed by the SNPs of *CPS1* rs715 and rs1047891 (p.Thr1406Asn) yield a protective association with decreased risk of coronary artery disease in women^35^. As mentioned in the introduction, those findings led to the speculation that individuals with the A-allele may have an advantage in terms of availability of the NOS substrate L-arginine, especially under conditions of environmental stress^15^,^23^,^26^. Accordingly, the serum levels of arginine were higher in term newborns carrying the AA genotype^15^. However, activity reports on the *in vitro* activity of the p.Thr1406Asn variants are contradictory^22^. Thus, natural (A-encoded) Asn1406 CPS has been reported to have higher enzymatic activity than the (C-encoded) Thr1406 variant^15^,^23^,^26^ but recombinant Asn1406 CPS1 showed inferior catalytic properties^36^. In order to evaluate whether p.Thr1406Asn genotypes correlated with urea cycle intermediates levels in preterm infants, we measured the concentrations of arginine and citrulline in the first 128 infants included in the present cohort and we did not observe any significant differences^28^. This lack of effect of *CPS1* genotype on L-arginine concentrations has been also reported in adults^25^. Nevertheless, one limitation of our study was that L-arginine levels were determined between 6 and 12 hours after birth, whereas NEC has its onset later in life^33^,^34^. It can be speculated that the alterations in arginine levels related to the p.Thr1406Asn polymorphism may be only relevant under the stress conditions generated around the time of NEC onset. At that moment, being carrier of a genetic variant that potentially increases NO production (i.e., the A-allele) might be of critical relevance because NO is a key regulator intestinal blood flow, protector of the mucosa, and modulator of the inflammatory response^10^,^11^,^12^. In addition, the infants homozygous for the C-allele might be more susceptible to NEC and arginine supplementation^16^,^20^,^21^ might be particularly indicated in this group.

One limitation of our study is that we did not collect information on the feeding practices. Human milk protects against NEC^1^,^2^,^3^ and, although the preterm formulas currently used have concentrations of arginine similar to the human milk^16^, arginine intake might be different depending on the infants diet. Plasma arginine levels are likely to represent a balance between arginine intake, arginine synthesis, and the demands of protein synthesis and the multiple metabolic pathways for arginine utilization^14^,^37^. Enteric arginine synthesis appears to be necessary to cover neonatal requirements, because mammalian milk is a relatively poor source of arginine, whereas its precursors proline and glutamine are abundant^38^. In fact, proline is the major contributor to arginine synthesis in human preterm infants^39^. CPS, ornithine aminotransferase, and argininosuccinate synthetase are key enzymes in the control of de novo intestinal synthesis of arginine, which are already expressed in the mid gestation human intestine^38^. However, hypoargininemia often develops in preterm infants, in particular if they are maintained on total parenteral nutrition, and it has been suggested that the intestine only produces arginine if substrate is supplied through enteral nutrition^14^,^37^,^38^,^39^. In situations of reduced availability of the substrate L-arginine or the cofactor BH_4_, NOS enzymatic activity becomes uncoupled, resulting in the production of superoxide instead of NO^40^. Therefore, besides resulting in a paucity of NO, the uncoupled enzyme will generate free radicals resulting in further intestinal damage. In addition, it should be taken into account that, besides NO, arginine is a substrate for synthesis of many biologically important molecules including agmatine, polyamines, and creatine^14^,^38^,^41^. These metabolites have roles in energy metabolism, gene expression, apoptosis, and cell proliferation and differentiation, which are crucial in intestinal homeostasis^14^,^38^,^41^. Our present results suggest that functional genetic variations in the CPS enzyme might be, at least partially, the link between hypoarginimemia and NEC in preterm infants^14^,^16^,^17^,^18^,^19^. Alternatively, recent experimental evidences highlighted the role of argininosuccinate lyase, another enzyme involved in the intestinal synthesis of arginine, in the pathogenesis of NEC^41^.

### Concluding remarks

NEC affects only a minority of preterm infants, which suggest an individual susceptibility toward the disease. Genetic polymorphisms might be an important factor in this individual susceptibility^2^,^4^,^5^,^9^,^42^,^43^,^44^,^45^,^46^,^47^,^48^,^49^,^50^,^51^. Our study provides further evidence that a functional variant of the *CPS1* gene may contribute to NEC susceptibility. Nevertheless, NEC is a complex multifactorial disease and an isolated genetic derangement may not be sufficient to account for the entire spectrum of its pathophysiology.

Given the importance of prematurity, intestinal function, immune defense, inflammatory signaling, and microcirculatory regulation mechanisms, potential variations in many genes could protect or leave a host infant susceptible to NEC^5^,^9^,^42^,^43^,^44^,^45^,^46^,^47^,^48^,^49^,^50^,^51^. Future studies investigating the association of multiple SNPs and NEC may allow for the development of a laboratory genetic test that could predict, when environmental factors are properly assessed, the risk/probability of preterm infants developing NEC and lead to more targeted therapies.

## Additional Information

**How to cite this article**: Moonen, R. M. *et al*. Association between the p.Thr1406Asn polymorphism of the carbamoyl-phosphate synthetase 1 gene and necrotizing enterocolitis: A prospective multicenter study. *Sci. Rep.* **6**, 36999; doi: 10.1038/srep36999 (2016).

**Publisher’s note:** Springer Nature remains neutral with regard to jurisdictional claims in published maps and institutional affiliations.

## Figures and Tables

**Table 1:** Baseline characteristics and neonatal complications in preterm infants with and without NEC.

	NEC –yes (n = 36)	*n* data missing	NEC – no (n = 441)	*n* data missing	*P* value
Birth weight (g)	844 (SD 216)	0	1016 (SD 266)	0	0.000
Gestational age (wks)	26.7 (SD 1.9)	0	27.9 (SD 1.9)	0	0.000
Male sex	17 (47.2)	0	238 (54.0)	0	0.419
Prenatal steroids	28 (80.0)	1	375 (88.2)	16	0.361
Preecclampsia	3 (8.6)	1	68 (15.6)	5	0.264
Chorioamnionitis	3 (8.3)	0	58 (13.3)	5	0.393
PROM	6 (16.7)	0	118 (27.2)	7	0.169
Vaginal delivery	17 (58.6)	0	147 (43.2)	2	0.042
Apgar (1 min)	5 [3–7]	1	6 [4–8]	5	0.026
Apgar (5 min)	8 [6–9]	1	8 [7–9]	6	0.114
RDS	30 (83.3)	0	378 (85.9)	1	0.671
Mechanical vent.	32 (88.9)	0	279 (64.0)	5	0.002
BPD	21 (58.3)	0	137 (31.3)	3	0.001
Hypotension	26 (72.2)	0	175 (40.1)	5	0.000
Sepsis	23 (67.6)	2	227 (51.7)	2	0.073
IVH	16 (44.4)	0	110 (25.1)	3	0.012
PVL	4 (11.1)	0	14 (3.2)	4	0.017
PDA	25 (71.4)	1	233 (53.2)	3	0.037
ROP	13 (41.9)	5	85 (20.5)	27	0.006
Mortality	8 (22.2)	0	42 (9.5)	0	0.017
Death before 34 wks	5 (13,9)	0	29 (6.6)	0	0.101

Results are expressed as mean (SD), median [interquartile range] or absolute numbers of patients (percentage). NEC: necrotizing enterocolitis (≥stage II); PROM: prolonged rupture of membranes; RDS: respiratory distress syndrome; BPD: bronchopulmonary dysplasia; IVH: intraventricular hemorrhage (≥grade 2); PVL: periventricular leukomalacia; PDA: patent ductus arteriosus; ROP: retinopathy of prematurity (≥stage II).

**Table 2:** Baseline characteristics and neonatal complications in preterm infants with and without the combined outcome NEC or death before 34 wks of corrected gestational age.

	NEC/death-yes (n = 65)	*n* data missing	NEC/death-no (n = 412)	*n* data missing	*P*-value
Birth weight (g)	818 (SD 230)	0	1032 (SD 261)	0	0.000
Gestational age (wks)	26.4 (SD 2.0)	0	28.1 (SD 1.8)	0	0.000
Male sex	34 (52.3)	0	221 (53.6)	0	0.841
Prenatal steroids	51 (81.0)	2	352 (88.7)	15	0.223
Preecclampsia	6 (9.4)	1	65 (16.0)	5	0.170
Chorioamnionitis	11 (17.2)	1	50 (12.3)	4	0.274
PROM	15 (23.8)	2	109 (26.8)	5	0.618
Vaginal delivery	34 (52.3)	0	142 (34.6)	2	0.006
Apgar (1 min)	5 [3–6]	1	6 [5–8]	6	0.000
Apgar (5 min)	8 [6–8]	1	8 [7–9]	6	0.000
RDS	54 (83.1)	0	354 (86.1)	1	0.513
Mechanical vent.	60 (92.3)	0	251 (61.7)	5	0.000
BPD	24 (37.5)	1	134 (32.7)	2	0.447
Hypotension	52 (80.0)	0	149 (36.6)	5	0.000
Sepsis	38 (60.3)	2	212 (51.7)	2	0.202
IVH	31 (48.4)	1	95 (23.2)	2	0.000
PVL	5 (7.8)	1	13 (3.2)	3	0.072
PDA	45 (71.4)	2	213 (52.0)	2	0.004
ROP	13 (25.0)	13	85 (21.6)	19	0.581

Results are expressed as mean (SD), median [interquartile range] or absolute numbers of patients (percentage). NEC: necrotizing enterocolitis (≥stage II); PROM: prolonged rupture of membranes; RDS: respiratory distress syndrome; BPD: bronchopulmonary dysplasia; IVH: intraventricular hemorrhage (≥grade 2); PVL: periventricular leukomalacia; PDA: patent ductus arteriosus; ROP: retinopathy of prematurity (≥stage II).

**Table 3:** Distribution of the *CPS1* p.Thr1406Asn polymorphism genotypes in preterm and term infants.

Population	CC	CA	AA	MAF
Total study group (n = 477)	248 (52.0)	176 (36.9)	53 (11.1)	0.296
Term control group (n = 96)^34^	52 (54.2)	34 (35.4)	10 (10.4)	0.281

Results are expressed as absolute numbers of patients (percentage).

CC denotes homozygosity for the C-encoded *CPS1* p.Thr1406Asn polymorphism variant; AA homozygosity for the A-encoded *CPS1* p.Thr1406Asn polymorphism variant; CA heterozygosity for *CPS1* p.Thr1406Asn polymorphism; MAF: minor allele frequency.

**Table 4:** Distribution of the *CPS1* p.Thr1406Asn polymorphism genotypes in preterm infants with and without NEC.

Model	Genotype	NEC-no (*n* = 441) *N* (%)	NEC-yes (*n* = 36) *N* (%)	OR (95% CI)	*P*-value	aOR (95% CI)	*P*-value
**Codominant**	CC	226 (51.2)	22 (61.1)	1 (reference)		1 (reference)	
	CA	163 (37.0)	13 (36.1)	0.82 (0.40–1.67)	0.59	0.87 (0.41–1.88)	0.73
	AA	52 (11.8)	1 (2.8)	0.20 (0.03–1.50)	0.12	0.21 (0.03–1.66)	0.14
**Dominant**	CC	226 (51.2)	22 (61.1)	1 (reference)		1 (reference)	
	AA_CA	215 (48.8)	14 (38.9)	0.67 (0.33–1.34)	0.26	0.71 (0.34–1.48)	0.36
**Recessive**	CC_CA	389 (88.2)	35 (97.2)	1 (reference)		1 (reference)	
	AA	52 (11.8)	1 (2.8)	0.21 (0.03–1.59)	0.13	0.23 (0.03–1.71)	0.15
**Additive**				0.64 (0.36–1.11)	0.11	0.66 (0.36–1.19)	0.17
**MAF**		0.303	0.208		0.09		

OR; odds ratio; aOR: odds ratio adjusted for birth weight, gestational age, Apgar score after 1 minute, mechanical ventilation, hypotension and PDA; CI: confidence interval; MAF: minor allele frequency; CC denotes homozygosity for the C-encoded *CPS1* p.Thr1406Asn polymorphism variant; AA homozygosity for the A-encoded *CPS1* p.Thr1406Asn polymorphism variant; CA heterozygosity for *CPS1* p.Thr1406Asn polymorphism.

**Table 5:** Distribution of the *CPS1* p.Thr1406Asn polymorphism genotypes in preterm infants with and without the combined outcome NEC or death before 34 wks of corrected gestational age.

Model	Genotype	NEC/death-no (*n* = 412) *N* (%)	NEC/death-yes (*n* = 65) *N* (%)	OR (95% CI)	*P*-value	aOR (95% CI)	*P*-value
**Codominant**	CC	206 (50.0)	42 (64.6)	1		1	
	CA	156 (37.9)	20 (30.8)	0.63 (0.36–1.11)	0.11	0.63 (0.33–1.20)	0.11
	AA	50 (12.1)	3 (4.6)	0.29 (0.09–0.99)	0.05	0.28 (0.08–1.02)	0.05
**Dominant**	CC	206 (50.0)	42 (64.6)	1		1	
	AA_CA	206 (50.0)	23 (35.4)	0.55 (0.32–0.94)	0.03	0.54 (0.29–0.99)	0.04
**Recessive**	CC_CA	362 (87.9)	62 (95.4)	1		1	
	AA	50 (12.1)	3 (4.6)	0.35 (0.11–1.16)	0.09	0.33 (0.10–1.18)	0.09
**Additive**				0.59 (0.38–0.91)	0.02	0.58 (0.36–0.93)	0.03
**MAF**		0.311	0.2		0.03		

OR; odds ratio; aOR:odds ratio adjusted for birth weight, gestational age, Apgar score after 1 minute, Apgar score after 5 minute, mechanical ventilation, hypotension and PDA; CI: confidence interval; MAF: minor allele frequency; CC denotes homozygosity for the C-encoded CPS1 p.Thr1406Asn polymorphism variant; AA homozygosity for the A-encoded CPS1 p.Thr1406Asn polymorphism variant; CA heterozygosity for CPS1 p.Thr1406Asn polymorphism.

**Table 6:** Distribution of the *CPS1* p.Thr1406Asn polymorphism genotypes in several neonatal outcomes.

Outcome		No. of infants	CC	CA	AA	P-value
Gestational age (wks)		477	27.7 (SD 2.0)	28.0 (SD 1.7)	28.1 (SD 1.9)	0.16
Birth weight (g)		477	984 (SD 269)	1018 (SD 260)	1043 (SD 277)	0.23
RDS	yes	408	205 (50.3)	158 (38.7)	45 (11.0)	0.14
	no	68	42 (61.7)	18 (26.5)	8 (11.8)	
BPD	yes	158	89 (56.3)	53 (33.5)	16 (10.1)	0.39
	no	316	157 (49.7)	122 (38.6)	37 (11.7)	
BPD or death before 36 weeks	yes	187	109 (58.3)	59 (31.6)	19 (10.2)	0.07
	no	288	137 (47.6)	117 (40.6)	34 (11.8)	
Hypotension	yes	201	103 (51.2)	77 (38.3)	21 (10.4)	0.84
	no	271	141 (52.0)	98 (36.2)	32 (11.8)	
Sepsis	yes	250	132 (52.8)	86 (34.4)	32 (12.8)	0.32
	no	223	113 (50.7)	89 (39.9)	21 (9.4)	
IVH	yes	126	68 (54.0)	42 (33.3)	16 (12.7)	0.53
	no	348	178 (51.1)	134 (38.5)	36 (10.3)	
PVL	yes	18	12 (66.7)	3 (16.7)	3 (16.7)	0.18
	no	455	233 (51.2)	173 (38.0)	49 (10.8)	
PDA	yes	258	144 (55.8)	86 (33.3)	28 (10.9)	0.14
	no	215	101 (47.0)	89 (41.4)	25 (11.6)	
ROP	yes	98	55 (56.1)	33 (33.7)	10 (10.2)	0.54
	no	347	173 (49.9)	132 (38.0)	42 (12.1)	
ROP or death	yes	129	76 (58.9)	40 (31.0)	13 (10.1)	0.13
before screening	no	324	157 (48.5)	128 (39.5)	39 (12.0)	

Results are expressed as absolute numbers of patients (percentage) or mean (SD).

CC denotes homozygosity for the C-encoded CPS1 p.Thr1406Asn polymorphism variant; AA homozygosity for the A-encoded CPS1 p.Thr1406Asn polymorphism variant; CA heterozygosity for CPS1 p.Thr1406Asn polymorphism. RDS: respiratory distress syndrome; BPD: bronchopulmonary dysplasia; IVH: intraventricular hemorrhage (≥grade 2); PVL: periventricular leukomalacia; PDA: patent ductus arteriosus; ROP: retinopathy of prematurity (≥stage II).
